# Supplementary material for: Deletion of ribosomal protein genes is a common vulnerability in human cancer, especially in concert with TP53 mutations
Source: EMBO Mol Med. 2017 Mar 6;9(4):498–507. doi: 10.15252/emmm.201606660 (PMC5376749; doi:10.15252/emmm.201606660)
Supplement: Supplementary file 1 — Expanded View Figures PDF [file EMMM-9-498-s001.pdf]

Expanded View Figures

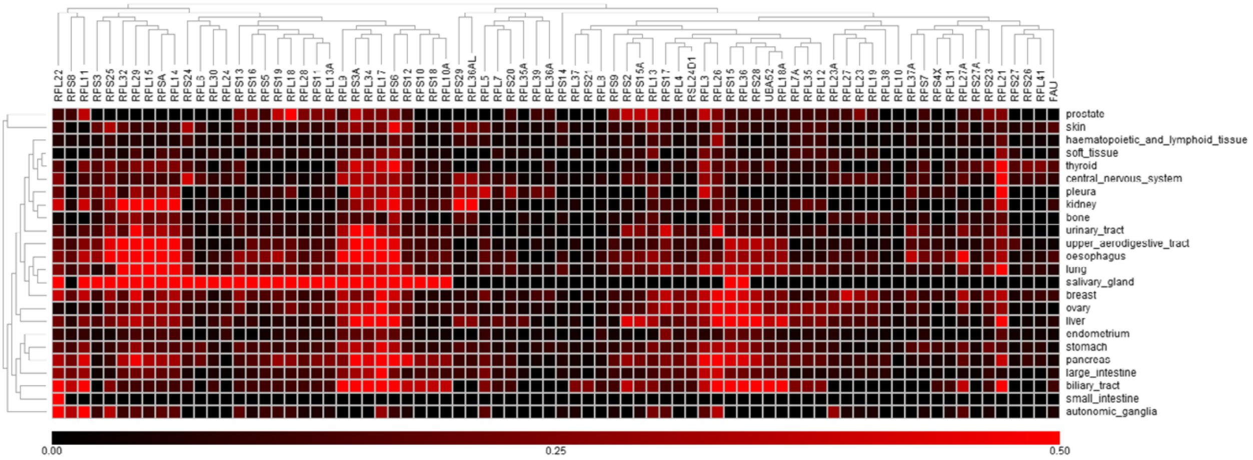

Figure EV1. RPG deletion frequencies in the CCLE data.

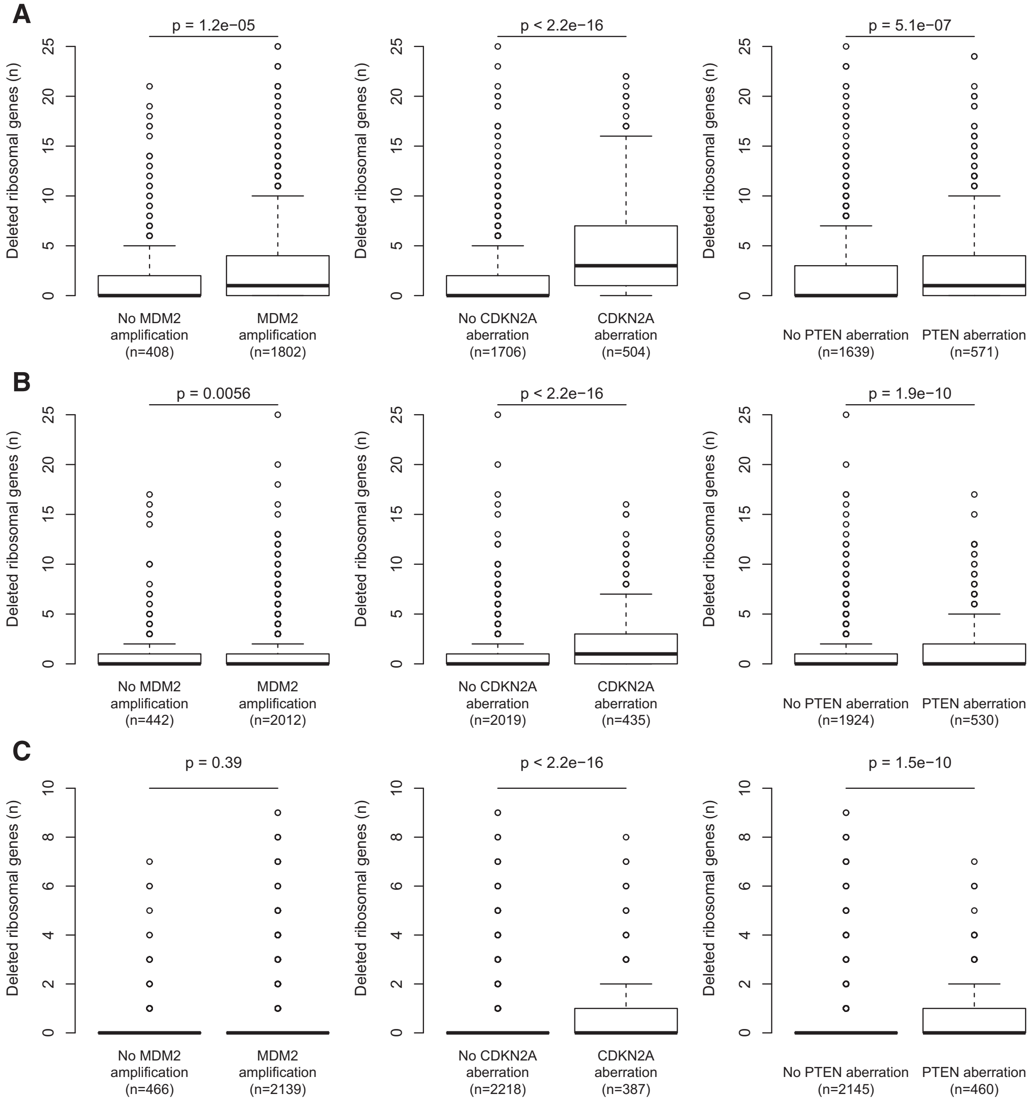

Figure EV2. Deletions of key genes of the p53 pathway in *TP53*-wild-type cases.

A–C Among tumors without detectable *TP53* deletion/mutation, the RPG deletions that could be detected were enriched in tumors harboring alternative p53-inactivating lesions, including *MDM2* amplifications, *CDKN2A* deletions, and *PTEN* deletions. Results obtained using $\log_2$ ratio thresholds (A) −0.3; (B) −0.5; (C) −0.7. To avoid biases introduced by co-deletion, RPGs on chromosome 12, 9, or 10 were excluded from the *MDM2*, *CDKN2A*, and *PTEN* analyses, respectively. Boxes indicate medians and the first and third quartiles. Whiskers indicate first and third quartiles ±1.5 times the interquartile range. Notches indicate confidence intervals around the median. *P*-values indicate significance by Wilcoxon rank-sum test.

© 2017 The Authors

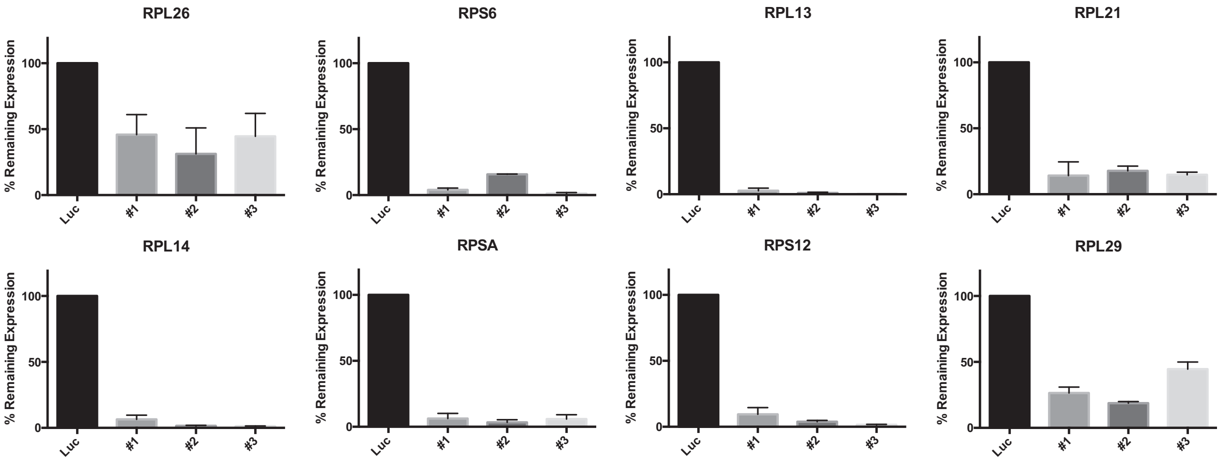

Figure EV3. Validation of shRNAs targeting frequently deleted RPGs.
mRNA expression levels of the indicated ribosomal protein gene were measured by quantitative real-time PCR. Ribosomal protein gene knockdown is shown as percentage of the gene expression in cells with control (luciferase) knockdown. Error bars are min-max of duplicates.

© 2017 The Authors

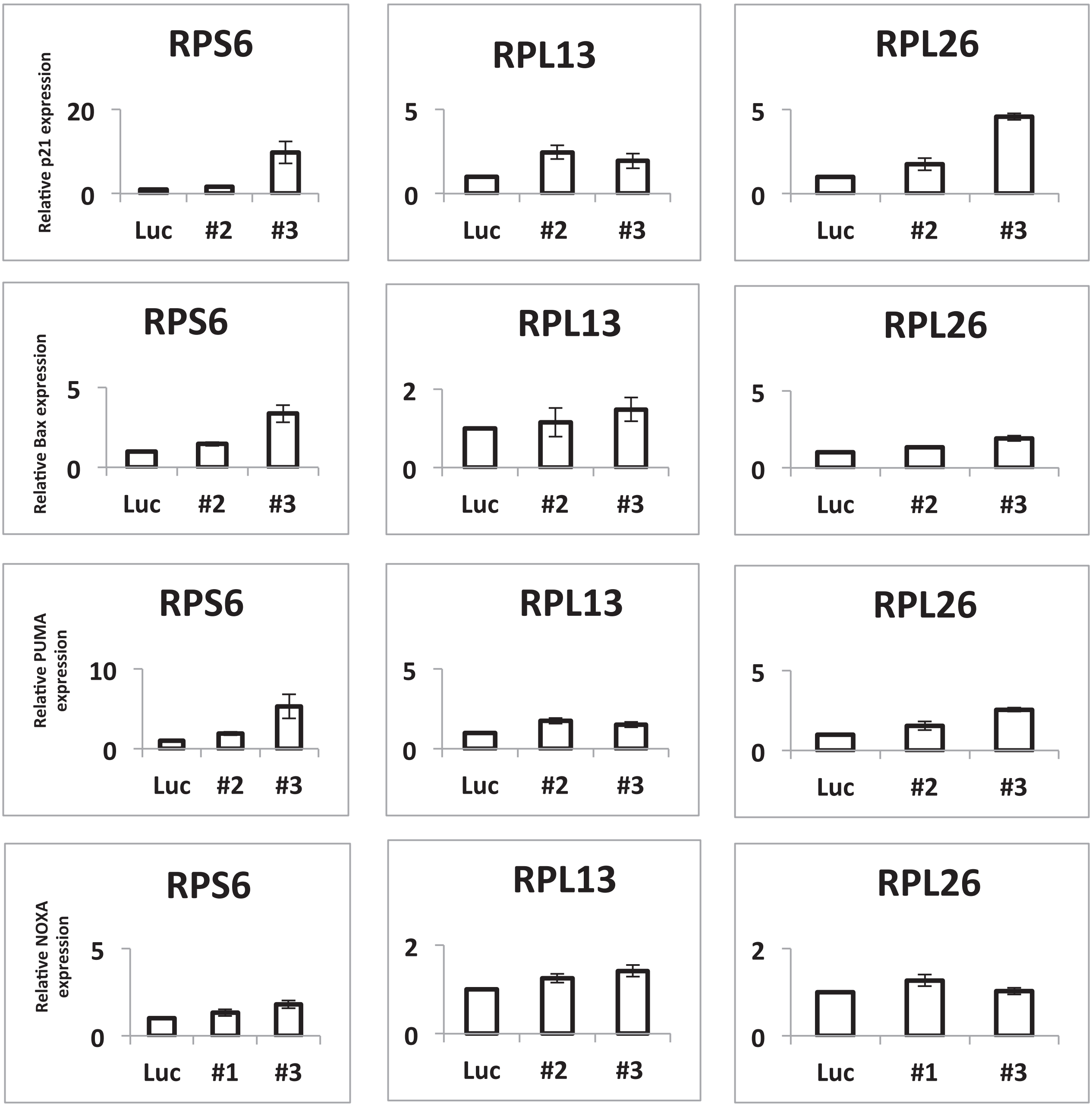

Figure EV4. Knockdown of identified RPGs induces p53 target genes.

Expression of the p53 target genes *P21*, *BAX*, *PUMA*, and *NOXA* in MOLM13 cells, as measured by qPCR after knockdown with shRNAs toward *RPS6*, *RPL13*, *RPL26* (two per gene; as in Fig 2), or luciferase (control). As shown, knockdown of these three RPGs induces the p53 target genes. Error bars are min-max. Duplicates.

 © 2017 The Authors

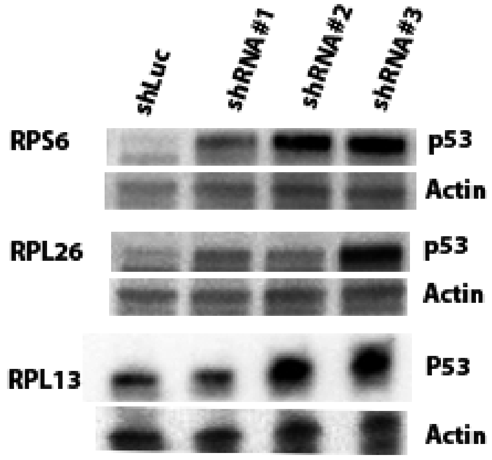

Figure EV5. Knockdown of identified RPGs induces p53.
Western blots showing p53 protein levels in MOLM13 leukemia cells after knockdown with shRNAs toward *RPS6*, *RPL26*, *RPL13* (three per gene), or luciferase (control).

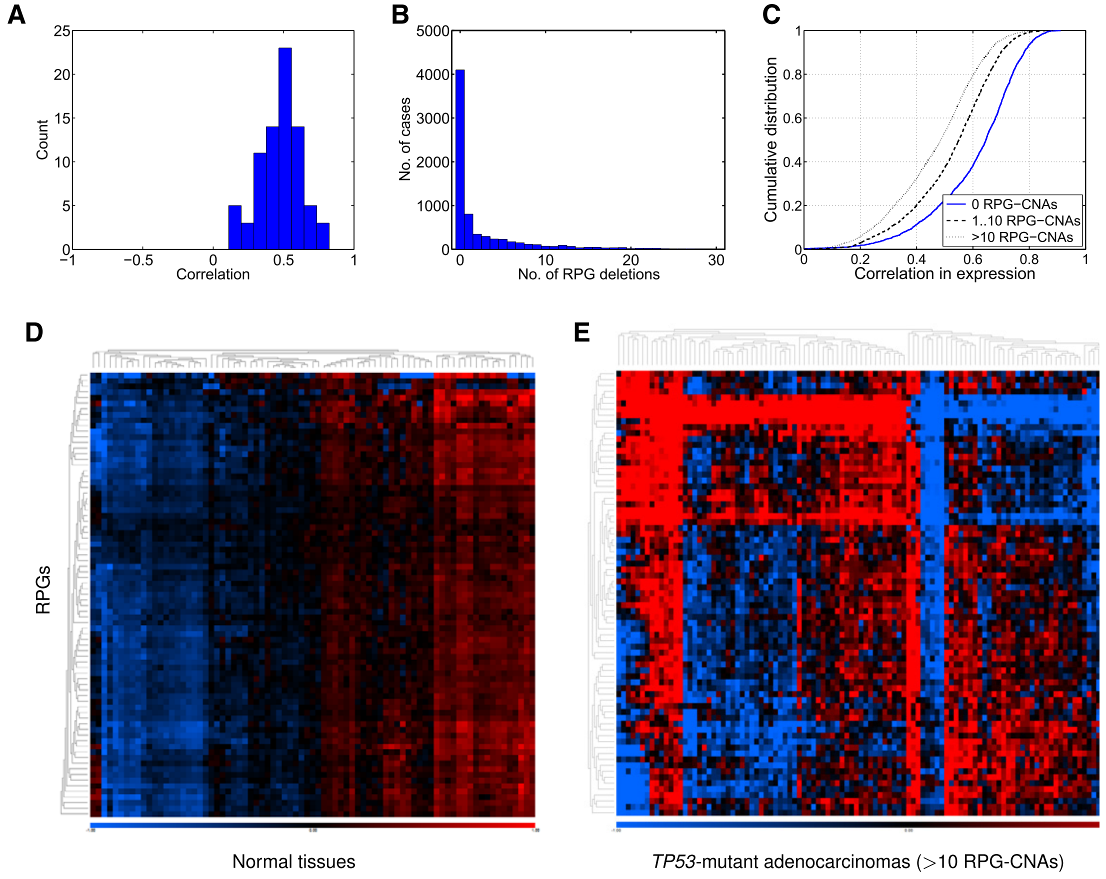

Figure EV6. While RPG are tightly co-expressed in normal tissues, copy number aberrations affecting RPGs lead to uncoordinated expression.

A Correlations between RPG copy number and expression across 4,919 TCGA samples.
B Numbers of RPG deletions per sample. Multiple RPG deletions are common.
C Cumulative distributions of Pearson correlation coefficient for all pairs of RPGs across the TCGA RNA-sequencing data. Tumors with more RPG copy number aberrations show less coordinated RPG expression.
D Expression of RPGs in normal tissues (SymAtlas; NCBI Gene Expression Omnibus GSE96).
E Expression of RPG in colorectal adenocarcinomas harboring 10 or more RPG copy number aberrations (TCGA samples).

© 2017 The Authors

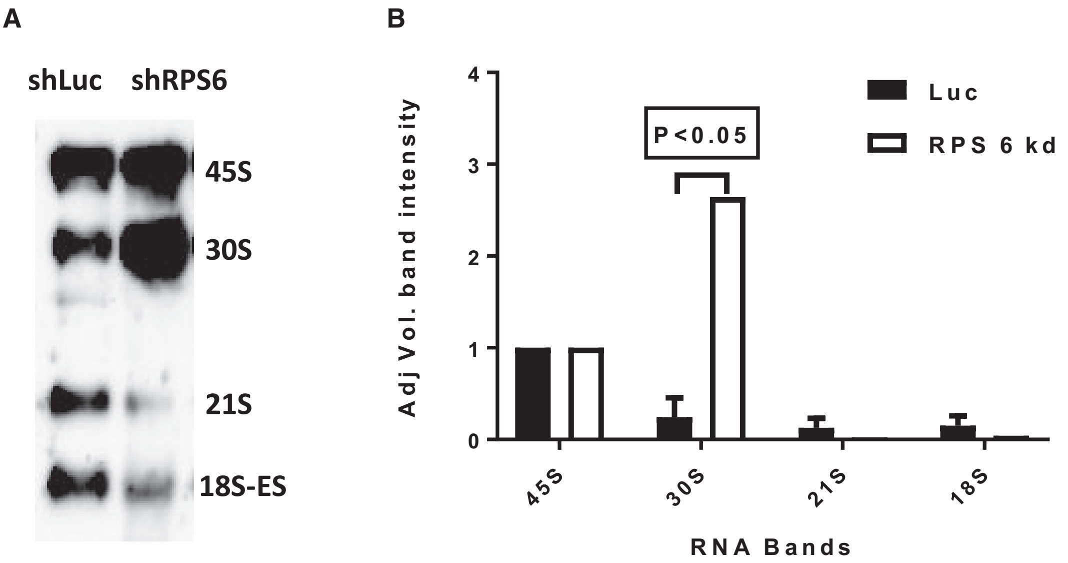

Figure EV7. Effects of *RPS6* knockdown on rRNA pattern.

A Representative Northern blot showing rRNA patterns in MOLM13 leukemia cells after knockdown with shRNA against RPS6 (shRPS6) or luciferase control (shLuc).

B Quantification of rRNA specifies from three Northern blot gels (error bars show standard deviation; Student's *t*-test *P*-value). As shown, knockdown of RPS6 yielded increased 30S rRNA levels and decreased 21S and 18S rRNA levels, which is congruent with the results obtained for primary ALL cells shown in Fig 3.

 © 2017 The Authors
